# Bifunctional chemokine-nanobody fusion protein enhances neutrophil recruitment to impede *Acanthamoeba* immune evasion

**DOI:** 10.1016/j.ebiom.2025.105685

**Published:** 2025-04-12

**Authors:** Zhenyu Wei, Jianlong Yuan, Qiankun Chen, Jinding Pang, Qingquan Shi, Bo Peng, Mingda Wei, Yuan Wei, Zhibao Zhang, Xinxin Lu, Xin Lin, Qingfeng Liang

**Affiliations:** a Beijing Institute of Ophthalmology, Beijing Tongren Hospital, Capital Medical University, Beijing Key Laboratory of Ophthalmology and Visual Sciences, Beijing, 100005, China; b Institute for Immunology, Tsinghua University School of Medicine, Tsinghua-Peking Center for Life Sciences, Beijing, China

**Keywords:** *Acanthamoeba* keratitis, Single-cell RNA sequencing, Neutrophil, Immune therapy

## Abstract

**Background:**

*Acanthamoeba* keratitis (AK) is a severe infectious disease that causes serious visual impairment and low quality of life. This study aims to investigate the immune landscape in AK, with the goal of improving treatment outcomes through immunotherapy.

**Methods:**

We conducted single-cell transcriptome sequencing on corneal tissues from nine patients (3 AK patients, 3 patients with fungal keratitis and 3 patients with bacterial keratitis). Bioinformatic analysis calculated the cell subsets and their proportions within different infectious keratitis. CellChat analysis elucidated the differential expression of chemokines in keratitis. After that, screening amebic nano-antibodies, synthesizing antibody-chemokine fusion proteins, and validated their affinity and chemotactic abilities *in vitro* and *in vivo*. And assessing of the therapeutic efficacy of antibody-chemokine fusion proteins.

**Findings:**

The UMAP plot demonstrated the 13 major cell clusters in infectious keratitis. Compared with non-AK group, the neutrophil proportion of AK group is markedly reduced. Cell communication indicated a diminished CXCL pathway in AK. *Acanthamoeba*-specific antibodies were obtained by screening a natural antibody library derived from alpacas. The amoeba-specific antibodies were conjugated with the CXCL1 chemokine, and this fusion protein exhibited robust binding affinity to *Acanthamoeba* and chemotactic capacity both *in vitro* and *in vivo*. Furthermore, *in vivo* animal investigations indicated that the fusion protein presented excellent therapeutic effect and could effectively eliminate the *Acanthamoeba* burden.

**Interpretation:**

This study revealed an immune evasion mechanism employed by *Acanthamoeba* and offered a therapeutic approach. It presents promising potential for enhancing the treatment of infectious diseases by targeting and overcoming challenges posed by immune evasion.

**Funding:**

This work was funded by 10.13039/501100001809National Natural Science Foundation of China (grant number 82171017 and 82471041) and the 10.13039/501100001809Beijing Municipal Public Welfare Development and Reform Pilot Project for Medical Research Institutes (PWD&RPP-MRI, JYY2023-6).

**Research in context**

**Evidence before this study**

Since the first identification of *Acanthamoeba* keratitis (AK) in 1974, its incidence has gradually increased in recent years. Despite substantial research efforts, the fundamental mechanisms underlying corneal infection remain poorly understand. This knowledge gap has contributed to limited treatment options and a poor prognosis. Previous investigators have revealed the correlation between immune cell (including neutrophil, macrophage and T cell) with prognosis. However, histopathology or *in vivo* confocal microscopy could only provide limited information on the selected section. As a result, there is an urgent need to comprehensively understand the host immune response in AK. Likewise, the potential of immune-based interventions as a therapeutic strategy should be further explored and evaluated.

**Added value of this study**

Our investigation reveals significant neutrophil depletion and downregulation of neutrophil-active chemokines in the cornea of patients with AK. The engineered immunotherapeutic modality successfully reconstituted the neutrophil-mediated anti-*Acanthamoeba* axis, demonstrating microenvironmental modulation capacity with therapeutic efficacy in preclinical models.

**Implications of all the available evidence**

Our study indicates that the antibody-cytokine fusion protein could effectively harness the targeting ability of antibodies and the chemotactic function of cytokines to recruit neutrophils. This immunotherapeutic approach holds promise not only for *Acanthamoeba* keratitis, but also for other infectious diseases characterized by immune evasion mechanisms.

## Introduction

*Acanthamoeba* keratitis (AK) is a severe ocular infectious disease caused by the *Acanthamoeba* parasite, and its incidence has surged in parallel with the growing use of corneal contact lenses, especially orthokeratology lenses.^1^, ^2^, ^3^, ^4^, ^5^, ^6^ Current treatment options primarily include antiparasitic medications as well as surgical interventions in severe cases.^7^, ^8^, ^9^, ^10^ However, these treatments are limited and often ineffective, due to the parasite’s invasive and insidious nature complicates therapeutic interventions.^11^ Despite extensive research, the immunological interactions between the host and the pathogen in AK remain poorly understood, leaving a gap in the understanding of the underlying mechanisms.

To investigate immune cell alteration in AK, we previously compared the corneal transcriptomes of AK patients with those of healthy controls. Surprisingly, the minimal differences in the composition of neutrophils and M1 macrophages were detected in both groups.^12^ This finding suggests that AK patients have an inadequate immune response to effectively kill the pathogens causing the infection, despite the presence of these inflammatory cells.^13^ There are several hypotheses including the alteration of surface antigens during the transformation of the parasite from trophozoites to cysts,^14^^,^^15^ the degradation of host antibodies by the parasite,^16^^,^^17^ or the parasite's modulation of the host immune response.^18^ However, these hypotheses are primarily derived from pathology or animal model results, while the precise corneal changes in the immune landscape and functions of immune cell in AK patients, as well as the methods to reverse this inadequate immune response, remain unclear.

To reveal the specific types and functions of immune cells in AK, single-cell RNA sequencing (scRNA-Seq) of infectious keratitis patients’ cornea, including bacterial keratitis (BK), fungal keratitis (FK), AK, was conducted. We defined the transcriptional profiles and immune landscape of infectious keratitis from patients’ cornea. Moreover, we highlighted significant alterations in immune cell recruitment and function, particularly in AK, where reduced neutrophil recruitment emerged as a potential factor in persistent infection. Building on these insights, we culminated in the development of a chemokine-nanobody fusion protein derived from *Acanthamoeba*-specific nanobody, which was screened from a phage display nanobody library derived from a pool of *alpaca* B cells. This study provided a valuable insight into the host immune response in AK, highlighting neutrophil abnormalities that contribute to its chronicity. Additionally, the innovative antibody-targeted chemokine therapy offers a promising strategy against immune-evasive infectious diseases, paving the way for new therapeutic avenues.

## Methods

### Patients enrolled and data collection

Approved by the Medical Ethics Committee of Beijing Tongren Hospital (TRECKY2021-024), this study was conducted at the Beijing Tongren Hospital from September 2022 to October 2023. All these participants were informed of the aims of the study and provided written informed consents in accordance with the declaration of Helsinki. The enrolled patients had a definite diagnosis of AK, FK, or BK. The diagnosis relied on clinical manifestations and laboratory tests, requiring at least one positive result from corneal scraping smears or microbial cultures. The samples from corneal scraping were placed on glass slides with Gram and Giemsa staining. Microbial cultures were conducted using blood agar, chocolate agar, potato dextrose, and Page's medium with inactivated *Escherichia coli* at the Department of Ocular Microbiology at the Beijing Institute of Ophthalmology. Exclusion criteria comprised: (1) patients with mixed ocular infections; (2) patients with a history of ocular infection, ocular inflammation, or ocular trauma which were unrelated to the current keratitis; (3) patients with chronic infections like Hepatitis B, C, and HIV.

Patient data were recorded following a standard protocol, including demographics, duration from symptom onset to diagnosis, risk factors, history of steroid use, preoperative and postoperative visual acuity of the affected eye, and surgical modality employed. Slit lamp and *in vivo* confocal microscopy (IVCM) images were acquired for all subjects. Slit lamp images were taken at 10× magnification using a calibrated Haag-Streit BX900 slit-lamp bio-microscope (Haag Streit AG, Bern, Switzerland). IVCM images were taken by the confocal microscope HRT3 (Heidelberg Engineering, Heidelberg, Germany) with 800× magnifications, and used for detecting *Acanthamoeba* trophozoite and cysts, fungal hyphae, and inflammatory cells.

For ethical considerations, corneal transplant surgeries were conducted solely for therapeutic purposes. So only corneal tissue in the advanced or late stage could be collected. The corneas were divided into two parts in a biological safety cabinet. Before the next steps, one was preserved in a tissue storage solution (Miltenyi, Cat# 130-100-008) at 4 °C. Usually, the corneas were stored and further dissociated within 4 h. Another part was used for immunohistochemistry.

### Preparation of single cell suspension, library construction, and sequencing

Following the protocol established by Ogawa et al.,^19^ a single-cell suspension of the infected cornea was prepared and filtered through a 40 μm cell strainer. Following centrifugation at 400*g* for 5 min, the cell pellet was incubated with RBC lysis buffer (BD Biosciences, Cat# 555899) on ice for 3 min. After washing twice, the cell pellets were stained with antibodies against 7AAD (BD Biosciences, Cat# 559925) and sorted using a BD Aria SORP instrument to minimize impurities. Cell number and viability were assessed using a fluorescence cell analyser (Shanghai RuiYu Biotech CO. Ltd., Shanghai, China). This method generated a single cell suspension with over 90% viability. Libraries were created using the Single Cell 3' Library (10× Genomics, Cat# 1000121) and Gel Bead Kit v3.1. Then the cDNA library was amplified and sequenced on an Illumina Novaseq 6000 sequencer (2 × 150bp).

### scRNA-seq data processing

Single-cell gene expression data were processed using the 10× Genomics Cell Ranger (v5.0.1), generating FASTQ files using ‘cellranger mkfastq’. The raw FASTQ files were processed with ‘cellranger count’. The reads were aligned to the human reference genome (GRCh38, v3.0.0), and the gene expression matrix was quantified using a unique molecular identifier (UMI) and cell barcode. The generated scRNA-seq lists were loaded, merged and preprocessed using R software (v4.2.2) with Seurat (v4.3.0). DoubletFinder was utilized to predict and remove doublet cells from each sample. Then cells were filtered according to read counts, gene numbers and mitochondrial gene counts. Cells with <200 or >5000 UMIs and with >10% mitochondrial reads were excluded.

### Clustering dataset

Following the exclusion of low-quality cells, the data underwent downstream analysis utilizing the Seurat pipeline. Normalization of raw counts was performed by the NormalizeData function with default parameters (method = "LogNormalize”, scale.factor = 10, 000). Genes were ranked in descending order based on residual variance using the “vst” method from the FindVariableFeatures function of Seurat for normalized data, identifying the top 2000 genes as highly variable genes (HVG). Then we applied the RunPCA function with default settings to reduce dataset dimensionality, scaled using the ScaleData function, focusing on HVG, and accounting for mitochondrial effect. Harmony was applied immediately after PCA for reducing the batch effect between samples. We constructed a Shared Nearest Neighbour Graph utilizing the top effective principal components and applied the Louvain algorithm for cell clustering. Subsequently, we employed the Uniform Manifold Approximation and Projection (UMAP) dimensional reduction technique for data visualization, resulting in 13 unsupervised cell clusters.

### Identification of signature genes of meta-clusters

Differential cell analysis for each cluster was conducted using the FindMarkers function with the default parameters of the Seruat package. Marker genes were identified using the Wilcoxon rank-sum test to detect each cluster by comparing them with all other experimental cells. Cell types were then assigned to these clusters and annotations were plotted with R package scRNAtoolVis (v0.0.7). The proportions of various cell types and subtypes were calculated accordingly. A Z-test was employed to analyse the proportion of cell subpopulations in different group. R package ClusterGVis (v0.1.1) enables a streamlined process to perform gene clustering, visualize the gene expression matrix and provides the GO analysis results corresponding to the genes within these clusters.

### Inference of cell–cell interaction

To better highlight the differences between AK and other infectious keratitis, we consolidated BK and FK into a non-AK group and compared it with the AK group. Intercellular signalling pathway patterns and intensities were predicted using the CellChat R package (v2.1.2). A normalization matrix was loaded and run for each group. We employed a human ligand-receptor interaction database to estimate the communication probability between cell groups within each specified group. The strength of the interaction was extracted from the inferred interaction weight, and the CellChat objects were then merged and visualized through a netVisual_bubble and netAnalysis_river functions to enable comparison across the groups. The *P* values are corrected using Benjamini-Hochberg to control the false discovery rate.

### Immunohistochemistry

Another half of cornea tissue was fixed in 4% paraformaldehyde overnight. It was then embedded in paraffin and sectioned at a thickness of 4 μm. The tissue sections were subjected to antigen retrieval using EDTA buffer. After quenching endogenous peroxidase activity with 3% H_2_O_2_ for 20 min, the sections were permeabilized and blocked. They were then incubated overnight at 4 °C with the following primary antibodies: rabbit anti-human MPO (Proteintech, Cat#22225-1-AP), CD4 (Abcam, Cat# ab133616), CD8a (Abcam, Cat#ab237709), and CD68 (Abcam, #ab213363). The sections were washed and incubated with HRP-conjugated goat anti-rabbit IgG (Abcam, #ab205718) for 1 h at room temperature. Haematoxylin was used for counterstaining. Finally, the stained sections were imaged using a microscope equipped with a digital camera (Olympus BX-51, Olympus, Tokyo, Japan).

### Pathogen culture

*Acanthamoeba* strains were isolated from the corneas of 3 AK patients. Of these, two individuals were infected after wearing orthokeratology lenses, and one person was infected due to trauma. The genotypes of the isolated *Acanthamoeba* strains were confirmed as T3, T4, and T11.^20^ *Acanthamoeba* were cultured in PYG medium at 30 °C until reaching the logarithmic growth phase. Trophozoites were then scraped off using a cell scraper, centrifuged at 1500 rpm for 5 min at 4 °C, washed 3 times with PBS and counted. Except for ELISA assay to detect binding affinity, all other experiments were conducted using the *Acanthamoeba* with T4 genotype.

The strains (*Pseudomonas aeruginosa*, *E. coli*, *Streptococcus oralis*, *Staphylococcus epidermidis*, *Staphylococcus aureus*, *Fusarium solani*, *Aspergillus fumigatus*, and *Candidi albicans)* were isolated from the cornea of patients with BK or FK at Beijing Tongren Hospital. These bacteria were cultured on the blood agar media at 35 °C for 24 h. A bacterial suspension was prepared by selecting a single colony and adjusting it to 1 × 10^7^ colony-forming units (cfu)/mL using sterile PBS. These fungi were cultured on potato dextrose agar at 28 °C for 4 days. The fungal suspension was measured by a cell counting chamber and adjusted to a concentration of 1 × 10^7^ cfu/mL in PBS.

### Establish mouse models with infectious keratitis

Ethical approval for all animal experimental procedures was provided by the Animal Ethics Committee of Beijing Tongren Hospital (Ethics No. TRLAWEC2024-40). All experiments used specific pathogen-free C57BL/6 mice, which were obtained from Charles River (Charles River, China). The mouse model with BK and FK was employed following established laboratory protocols.^21^^,^^22^ The AK mouse model was established through intrastromal injection of *Acanthamoeba*, following the method outlined previously.^23^ Mice were anesthetized via intraperitoneal injection of ketamine and xylazine. Proparacaine hydrochloride was applied to the cornea for topical anaesthesia. Parasites were suspended in PBS at a concentration of 1 × 10^7^ cells/ml. After epithelial abrasion, 1 μL of the suspension (containing 1 × 10^4^ *Acanthamoeba*) was injected into the stroma using a 33-gauge Hamilton syringe.

Slit-lamp microscopy was utilized to examine the eyes, and infection severity was evaluated using a previously scoring system.^8^ In brief, corneal evaluations were categorized as follows: Score 0 - normal cornea without infection, score 1 - partial corneal opacity covering the pupil, score 2 - dense corneal opacity covering the pupil, score 3 - dense opacity covering the entire anterior segment, and score 4 - corneal perforation. Similarly, IVCM images was also obtained on the mice.

### ELISA

To determine the CXCL1 expression level, different infection mouse models (n = 3 per group) were prepared as described before. On the third day after infection, mice were sacrificed to obtain the corneas. Homogenates of corneal samples were centrifuged, and supernatant was used for analyses. The concentrations of mCXCL1 were measured using the ELISA kit (R&D Systems, Cat# MKC00B).

### VHH antibodies screening from alpaca naïve phage display library

The alpaca naïve phage display library, consisting of approximately 5 × 10^12^ cfu (BriSTAR Immunotech Biotechnology Co., Ltd., Beijing, China), was utilized for antibody screening. The VHH library in *E. coli* strain TG1 was infected with the helper phage M13KO7 (BriSTAR Immunotech Biotechnology Co., Ltd., Beijing, China) to generate phages displaying the encoded VHHs as fusion proteins. *Acanthamoeba castellanii* with T4 genotype used for each panning round.

Following three panning rounds, 96 individual clones were selected, inoculated into 2 × YT medium with 100 μg/mL ampicillin (Solarbio, Cat# A8180) and kanamycin (Solarbio, Cat# K8020), and incubated overnight at 37 °C. *Acanthamoeba* was utilized as the positive control, and a the 3% BSA solution was used as the negative control. Following a 1-h incubation, the cell-free phage supernatant was assessed by phage-ELISA using a mouse anti-M13-HRP antibody (SinoBiological, Cat# 11973-MM05T-H). Clones with a P (positive OD450-OD630)/N (negative OD450-OD630) ratio greater than 2 were considered positive. The positive candidates were then sequenced (Tsingke) and their complementarity-determining region (CDR) amino acid sequences were aligned. Clones with unique sequences were further evaluated by phage-ELISA against bacteria, fungi, and *Acanthamoeba* with the T3 and T11 genotypes, which are commonly associated with ocular diseases. The method of these pathogens culture has been previously described. Only the clones that showed positive results with *Acanthamoeba* of all genotypes and negative results with all bacteria and fungi were selected for the next step.

### Protein structure prediction

Structural predictions were conducted using AlphaFold 2. For each prediction, five models were generated and optimized using the AMBER force field. Only the model rated as rank 1 is presented in the resulting images.

### Expression and purification of VHHs in *E. coli*

Monovalent VHH nanobody sequences and sequence of nanobody carrying mCXCL1 were synthesized by Biointron (Taizhou, China) and subcloned into the pET-SUMO expression vector, featuring a N-terminal His-tag. The recombinant plasmids were transformed into *E. coli* BL21(DE3) cells. A 1 L culture was cultivated in 2 × YT medium with 100 μg/mL ampicillin at 37 °C and 220 rpm until reaching an OD600 of 0.6. Following this, 1 mM IPTG was added, and the culture was incubated at 16 °C and 220 rpm for an additional 16 h. Cells were collected via centrifugation at 7000 rpm for 5 min and then resuspended in a lysis buffer (50 mM Tris, 300 mM NaCl, 1% Triton X-100, pH 8.0). The mixture was centrifuged at 11,000 rpm for 20 min to remove cell debris. The nanobody underwent purification through nickel affinity chromatography, as previously outlined, was subsequently processed using size exclusion chromatography with SRT-C SEC-300 5 μm column (Sepax). Nanobody fractions were concentrated to about 1 μg/μl with 10% glycerol, rapidly frozen in liquid nitrogen, and stored at −80 °C for later use. SDS-PAGE was used to evaluate the quality of the purified proteins.

### Immunofluorescence staining

To assess antibody binding affinity, the suspended *Acanthamoeba* were applied to a polylysine coated microscope slide, dried, washed thrice with PBS, and air-dried again. The primary antibodies were switched to Acab95 and Acab95-CXCL1 which expressed by *E. coli*. The secondary antibody was anti-his-CoraLite® Plus 488 (Proteintech, Cat# CL488-66005). After DAPI staining, the slides were then imaged using a fluorescence microscope (Olympus DP72, Tokyo, Japan).

Corneal slides were deparaffinized using xylene and rehydrated through a series of graded ethanol to PBS. Antigen retrieval was performed by pressure cooking the samples in EDTA buffer. Since Acab95 and Acab95-CXCL1 have been injected into cornea, they were directly stained with anti-his-CoraLite® Plus 488 and DPAI. The remaining procedures were conducted similarly to the previously described immunofluorescence staining of slices.

### Flow cytometry

To detect the binding affinity of antibody, expressed protein incubated with *Acanthamoeba* for 1 h. Beyond Acab95, Acab18 also was expressed in the research. Because the similar molecular weight and the low binding affinity, it was used as the fake-antibody to exclude false positive. After Fc-blocking and washing, *Acanthamoeba* were incubated with anti-his-AF647 antibody (BioLegend, Cat# 652513). All incubation steps were on ice. Samples were examined using the Attune NxT flow cytometer (Thermo scientific). FlowJo software (v10.8.1) was used to analyse flow data.

### Neutrophil isolation

The whole blood of mice was obtained by extracting the eyeball blood under anaesthesia and loaded into heparinized anticoagulation tubes. Neutrophils were isolated according to the manufacturer's protocol of EasySep™ Mouse Neutrophil Enrichment Kit (StemCell, Cat# 19762). Neutrophils were labelled with Anti-CD11b-AF488 and incubated on ice for 30 min. After washing with 2% FBS/PBS, neutrophils were analysed by flow cytometry. Only these with over 95% purity were used for subsequent procedures.

### Transwell cell migration assay

To assess chemotactic ability, mouse neutrophils were positioned in the upper chamber of an 8 μm pore transwell. The neutrophil chemoattractant and PBS were added to the lower chamber, separately. The final concentration of were 0.11 pg/μl for mCXCL1 (Novoprotein, Cat# P12850) and 0.23 pg/μl for Acab95-CXCL1. After a 2h incubation, the number of neutrophils in each lower chamber was counted.

### Immune therapy

After the AK model was established (on day 0), 1 μL of the test compound was administered into the corneal stroma using a 33-gauge Hamilton syringe, under both general and topical anaesthesia, as previously described. The test compounds were: 0.11 pg/μL mCXCL1 (low-dose group), 110 pg/μL mCXCL1 (high-dose group), or 0.23 pg/μL Acab95-CXCL1 (Acab95-CXCL1 group). As a control, the same volume of PBS or Acab95 was injected.

### Quantitative PCR

To examine the pathogen load in cornea, mice were killed at different days and their corneas were obtained. Corneas were snap frozen and ground using a tissue lyser (Ningbo SCIENTZ Biotech Co, Ltd., Ningbo, China). DNA was extracted with the QIAamp DNA mini kit (Qiagen, Cat# 51304). Primers and probes specific for *Acanthamoeba* (Forward: CGACCAGCGATTAGGAGACG; Reverse: CCGACGCCAAGGACGAC; Probe: 5’-6FAM-TGAATACAAAACACCACCATCGGCGC-BHQ1-3’). Primer sequences and the PCR protocol were referred the study by Yoshifumi et al.^24^ A standard curve was established using a dilution series of known quantities of *Acanthamoeba castellanii* with T4 genotype, isolated from clinical patients.

### Haematoxylin and eosin staining

After 1-, 3-, 5- and 7-day post treatment, the eyeballs of AK mouse models were removed and fixed in a fixative solution for approximately 24 h (n = 3 for each time point; n = 24 total). The samples were then embedded in paraffin wax and sectioned. Tissue sections were mounted on slides and stained with haematoxylin and eosin (HE). Finally, the stained samples were imaged under an optical microscope.

### Statistical analyses

In addition to the algorithms described above, all other basic statistical analysis was performed in the R statistical environment. Data normality was evaluated using the Shapiro–Wilk test. For two-group comparison, if data follow a normal distribution, two-tailed Student's t-test was used. Nonparametric Wilcoxon rank sum test was applied to data that didn’t follow a normal distribution. Differences of *P* < 0.05 were statistically significant. Significance levels are indicated as follows: ∗ for *P* < 0.05, ∗∗ for *P* < 0.01, ∗∗∗ for *P* < 0.001.

### Role of funding source

This work was funded by National Natural Science Foundation of China (grant number 82171017 and 82471041) and the Beijing Municipal Public Welfare Development and Reform Pilot Project for Medical Research Institutes (PWD&RPP-MRI, JYY2023-6). These fundings had no role in the study design, data collection, data analyses, or data interpretation and were only used to purchase research equipment and reagents.

## Results

### Immune cell landscape of infectious keratitis

**Fig. 1:**
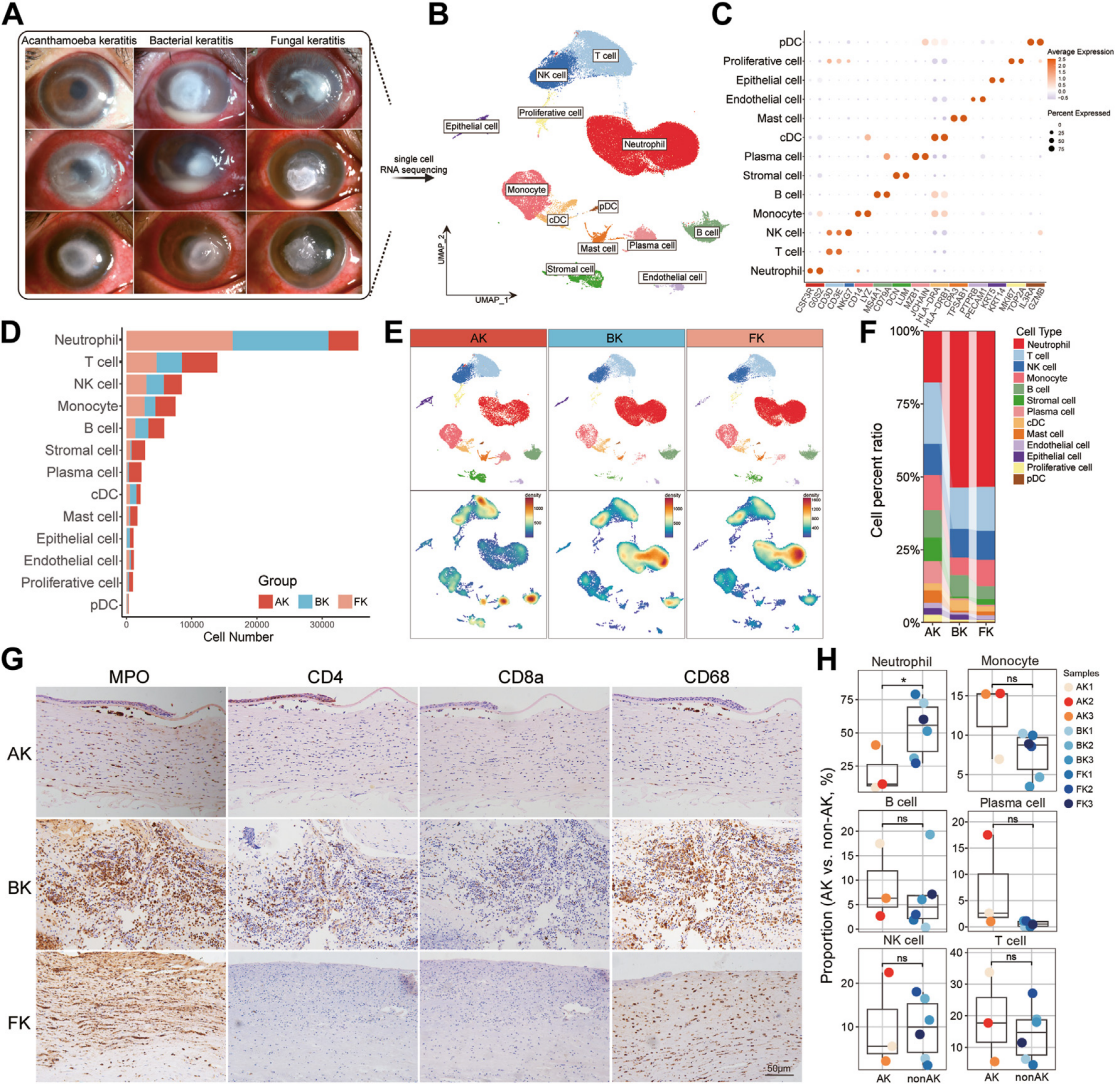
Major cell types in various infectious keratitis identified by scRNA-Seq. (A) Slit-lamp examination of corneas from patients with infectious keratitis. (B) UMAP depicting major cell lineages. (C) Bubble plot depicting expression levels of representative cell markers across clusters. (D) Cell composition of corneas in cases of infectious keratitis. (E) UMAP visualizations depict cell clusters (upper panels) and cell density (lower panels) among various disease groups. (F) Stacked bar plot depicting the variation in relative proportion of major cell types across different disease conditions. (G) Immunohistochemical staining of corneal tissue from patients with different infectious keratitis. (H) The proportions of representative cell types between AK (n = 3) and non-AK groups (n = 6, Z-test).

This study utilized scRNA-seq to examine the immune response in corneal infections across 9 patients, including AK, BK, and FK, with three patients in each group. Representative slit-lamp images of these patients were shown in Fig. 1A. The mean ages were 51.7 (47.5–59.0) years in the AK group, 52.0 (49.0–59.0) years in the FK group, and 31.9 (20.9–47.5) years in the BK group. Statistical analysis indicated no significant differences in age (*P* = 0.357) or gender (*P* = 1.000) between the groups. Table 1 summarizes basic information of patients, including symptom duration, risk factor, surgical procedure and so on.

**Table 1:** Demographics and clinical characteristics of patients.

Parameter	AK	FK	BK
Gender (Male/Female)	2/1	1/2	2/1
Age (years)	51.7 (47.5–59.0)	52.0 (49.0–59.0)	31.9 (20.9–47.5)
Duration (days)	50.0 (23.5–50)	45.0 (22.5–60)	16.0 (9.0–22.5)
Risk factor			
Ocular surface injury	1 (33.3%)	3 (100.0%)	2 (66.7%)
Agricultural	–	2 (66.7%)	–
Dust	1 (33.3%)	1 (33.3%)	–
Contact lens use	1 (33.3%)	–	–
Tap water exposure	1 (33.3%)	–	–
Unknown	–	–	1 (33.3%)
Visual acuity (decimal)			
>0.3	–	1 (33.3%)	–
0.02–0.3	2 (66.7%)	1 (33.3%)	1 (33.3%)
<0.02	1 (33.3%)	1 (33.3%)	2 (66.7%)
Scraping (+/−)	3/0	3/0	3/0
Culture (+/−)	2/1	2/1	2/1
*Acanthamoeba* spp.	2 (66.7%)	–	–
*Fusarium* spp.	–	1 (33.3%)	–
*Alternaria* spp.	–	1 (33.3%)	–
*Pseudomonas aeruginosa*	–	–	1 (33.3%)
*Streptococcus pneumoniae*	–	–	1 (33.3%)
HRT (+/−)	3/0	3/0	–
Surgical procedure			
Penetrating keratoplasty	2 (66.7%)	3 (100.0%)	2 (66.7%)
Deep anterior lamellar keratoplasty	1 (33.3%)	–	1 (33.3%)

After removing doublets and low-quality cells, 83,669 cells were eligible for further analysis. Of these, 25,846 cells (30.9%) were from the AK group, 30,470 cells (36.4%) from the FK group, and 27,353 cells (32.7%) from the BK group. UMAP was employed to visualize the cell populations (Fig. 1B), leading to the identification of 13 cell clusters. Using multiple cell-type reference datasets and canonical markers, we identified the following cell types: corneal epithelial (KRT5^+^, KRT14^+^), corneal stromal (DCN^+^, LUM^+^), corneal endothelial (PTPRB^+^, PECAM1^+^), proliferative cell (MKI67^+^, TOP2A^+^), neutrophil (CSF3R^+^, G0S2^+^), monocyte (CD14^+^, LYZ^+^), T cell (CD3D^+^, CD3E^+^), NK cell (NKG7^+^), B cell (MS4A1^+^, CD79A^+^), plasma cell (MZB1^+^, JCHAIN^+^), conventional dendritic cell (HLA-DRA^+^, HLA-DRB1^+^), plasmacytoid dendritic cell (IL3RA^+^, GZMB^+^), and mast cell (CPA3^+^, TPSAB1^+^) (Fig. 1C). The gene heatmap and GO enrichment analysis results for these cell subsets were presented in Supplementary Fig. S1. The top 100 marker genes were listed in Supplementary Table S1.

The bar graph suggests that neutrophils (42.5%) are the most prevalent, followed by T cells (16.6%), NK cells (10.1%), monocytes (9.0%), and B cells (6.9%) (Fig. 1D). The density map reveals that BK and FK are similar, while regions of high density in AK are predominantly concentrated around T cells, mast cells, B cells, and plasma cells (Fig. 1E). In both BK and FK groups, neutrophils accounted for the highest proportion of immune cells (53.6% in BK and 53.4% in FK). Subsequently, T cells (14.2% in BK and 15.1% in FK) and NK cells (9.8% in BK and 9.9% in FK) occupy the second and third largest category. In the BK group, the fourth and fifth ranked cell populations are B cells (7.3%) and monocytes (6.0%), respectively. In the FK group, the order is reversed, with monocytes (9.1%) ranking fourth and B cells (4.4%) ranking fifth. Notably, the AK group does not exhibit a significantly dominant cell subgroup; T cells (21.0%) constituted the largest proportion, followed by neutrophils (17.7%), monocytes (12.0%), NK cells (10.7%), B cells (9.4%), and plasma cells (7.5%). The overall distribution was shown in Fig. 1F. Immunohistochemical analysis of the patient's corneal tissue confirmed the differential distribution of cell types, particularly highlighting the marked neutrophil deficiency in AK (Fig. 1G).

According to the results of the correlation analysis (Supplementary Fig. S2), the BK and FK groups are combined into the non-AK group. The box plot indicates that only neutrophils exhibited statistically significant differences between the AK group and non-AK group (*P* = 0.029; Fig. 1H). Additionally, differential gene analysis was performed between the two groups (Supplementary Fig. S3). Neutrophil showed 63 upregulated genes in AK group and 27 upregulated genes in non-AK group. Based on differential genes of neutrophil, the GO functional enrichment and KEGG pathway enrichment results were obtained (Supplementary Fig. S4). GO terms mostly included ribosome-related pathway and KEGG enrichment only included the pathway of ribosome and coronavirus in AK group. In non-AK group, GO terms showed significant association with cytokine, including IFN-γ, IL-1 and IL-2. KEGG enrichment results also revealed the importance of chemokine signalling pathway.

### Chemotactic factor in mediating neutrophil infiltration

**Fig. 2:**
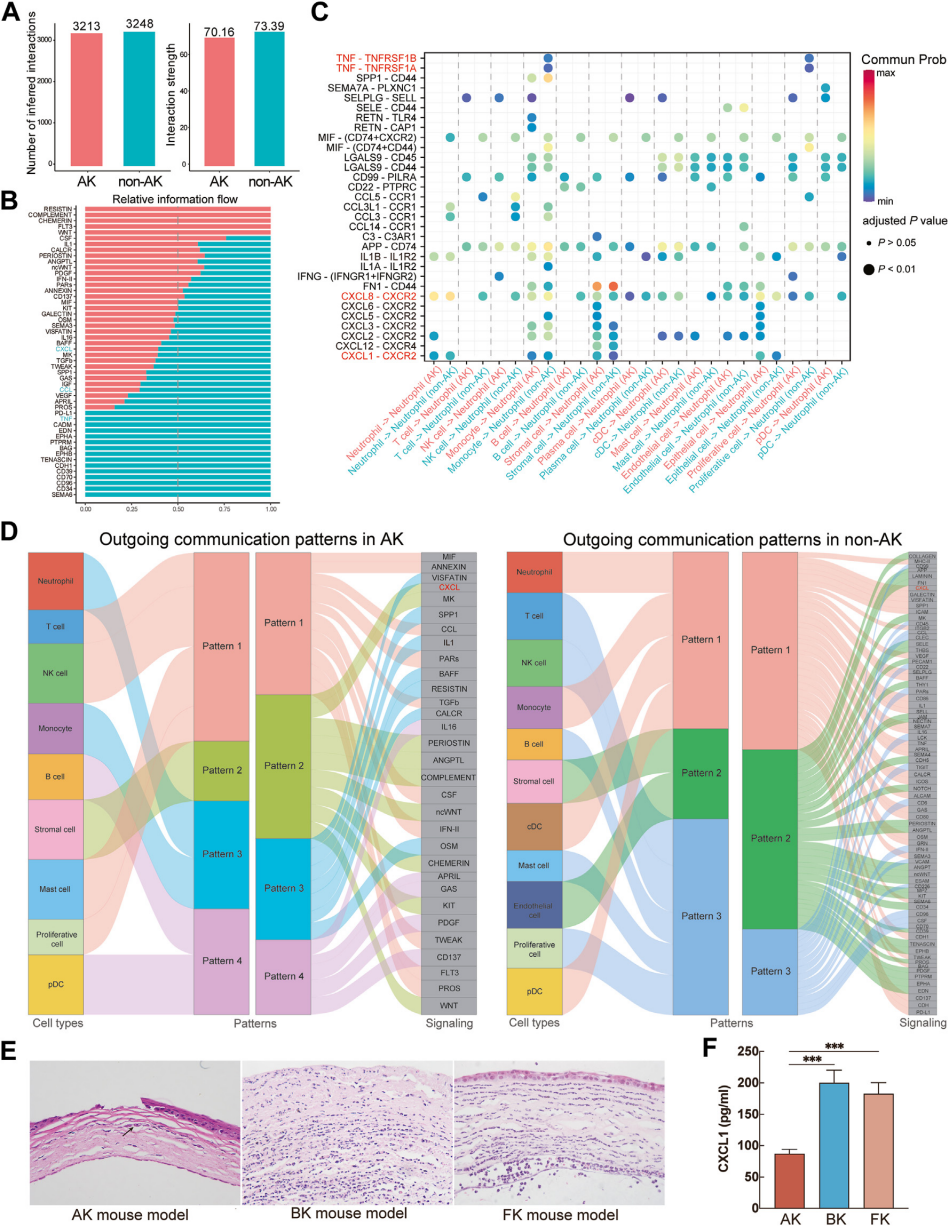
Comparison of cell communication between AK and non-AK group and validation the decreased chemokine secretion in mouse model. (A) Number of inferred interactions between AK and non-AK groups. (B) Bubble plot showing cell communication pattern among granulocyte and other cell clusters. Each dot signifies a ligand-receptor pair, with its size indicating the pathway involvement *P*-value and its colour representing the communication probability. (C) Overall information flow within each signalling pathway across the two groups. (D) Inferred outgoing communication patterns, demonstrate the relationship between signal patterns and cell groups, along with signalling pathways. The flow thickness represents the contribution of the cell group or signalling pathway to signal pattern. (E) HE examinations of corneal sections in each group 24 h after infection in each group (Black arrow: *Acanthamoeba*). (F) CXCL1 production measured by ELISA in mice after infection (n = 3 per group, n = 9 total; t-test). (∗∗∗ for P < 0.001).

We constructed a putative cell–cell interaction network by mapping interaction pairs onto cell subsets to clarify differences in cell–cell interactions. Overall, both the number and strength of the interactions among subclusters were decreased in the cornea from AK patients compared to those with other types of infectious keratitis (Fig. 2A). The relative information flow of each signalling pathway was assessed, revealing that only a small fraction of pathways (e.g., WNT, CSF, and IL-1) showed significant enhancement. While some pathways (e.g., CXCL, CCL, and TNF) were attenuated in the AK group, which may impact the chemotaxis of neutrophils (Fig. 2B).

Further analysis of ligand–receptor pairs revealed that the TNF-TNFRSF1A and TNF-TNFRSF1B interactions between monocytes and neutrophils were markedly increased in the non-AK group. Notably, the CXCL8–CXCR2 interaction between all cell clusters and neutrophils was also enhanced in the non-AK group, highlighting a crucial neutrophil chemotaxis pathway (Fig. 2C). Integration of output signals from different cell types revealed a distinct difference between the non-AK and AK groups. In the non-AK group, the outgoing communication among neutrophils, monocytes, cDCs, and pDCs formed a unified pattern that included CXCL signalling. In contrast, in the AK group, the CXCL signalling was isolated within the stromal cell compartment in a separate pattern (Fig. 2D). Based on these results, we propose that CXCL signalling is a critical pathway for neutrophil chemotaxis, a function impaired in the AK group. To validate our findings, we established animal models of various infectious keratitis. Histopathological examination of the cornea sections stained with HE indicated that the modelling results closely resembled human corneal infections (Fig. 2E). ELISA results suggested significant variations in CXCL1 expression among different infections on the third day post-infection, particularly with a pronounced decrease observed in the AK group (Fig. 2F).

**Fig. 3:**
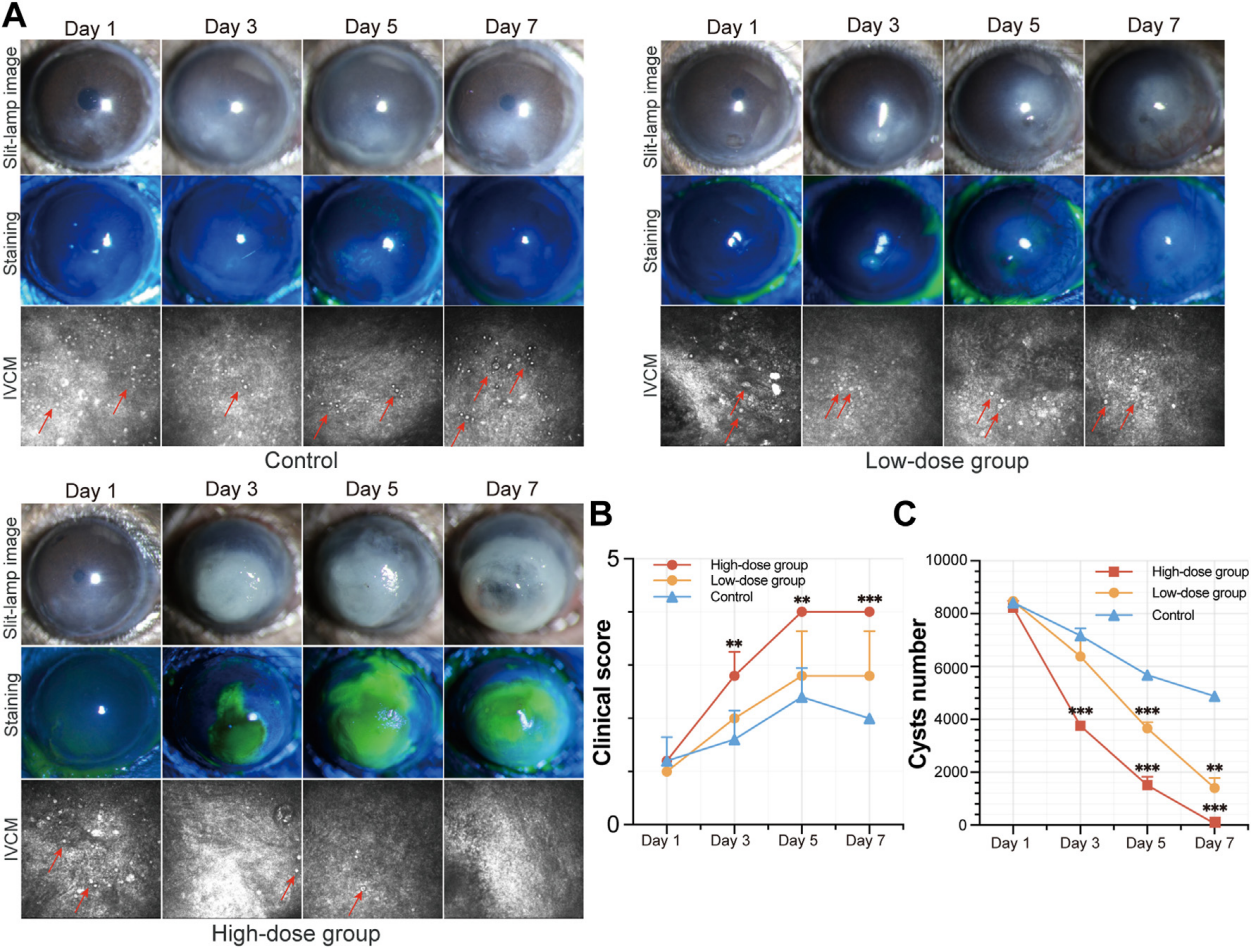
Clinical score and cysts number after different concentrations of chemokine therapy for AK model. (A) Slit-lamp microscope images, with and without fluorescein staining, and IVCM images of mice at different days post-treatment in different groups (Red arrow: *Acanthamoeba* cysts). (B) Clinical scoring of corneal condition (n = 3 per group, n = 9 total; t-test). (C) Quantification of *Acanthamoeba* cysts over time using real-time PCR (n = 4 for each time point, n = 48 total; t-test). (∗∗ for *P* < 0.01, ∗∗∗ for *P* < 0.001).

Therefore, we chose to complement CXCL1 in AK model. Although there were no significant differences in clinical scores between the low-dose CXCL1 treatment group and the PBS-treated control group, CXCL1 supplementation resulted in a notable reduction in corneal cyst burden by day 5. When high doses of CXCL1 were administered, a significant increase in clinical scores was observed starting from day 3, accompanied by a marked reduction in the corneal cyst burden on the same day. By day 7, the cyst burden within the cornea had approached nearly zero. IVCM also confirmed the reduction in amoebic burden within the cornea. However, significant corneal dissolution, nearing perforation, was also observed (Fig. 3A–C). These findings suggest that CXCL1 supplementation can be beneficial, but it must be administered at a low dose and with the ability to target the deeper corneal layers. The high-dose CXCL1 treatment, while effectively reducing the cysts burden, also led to corneal damage that could potentially result in perforation.

### Screening and identification of VHH antibodies targeting *Acanthamoeba*

**Fig. 4:**
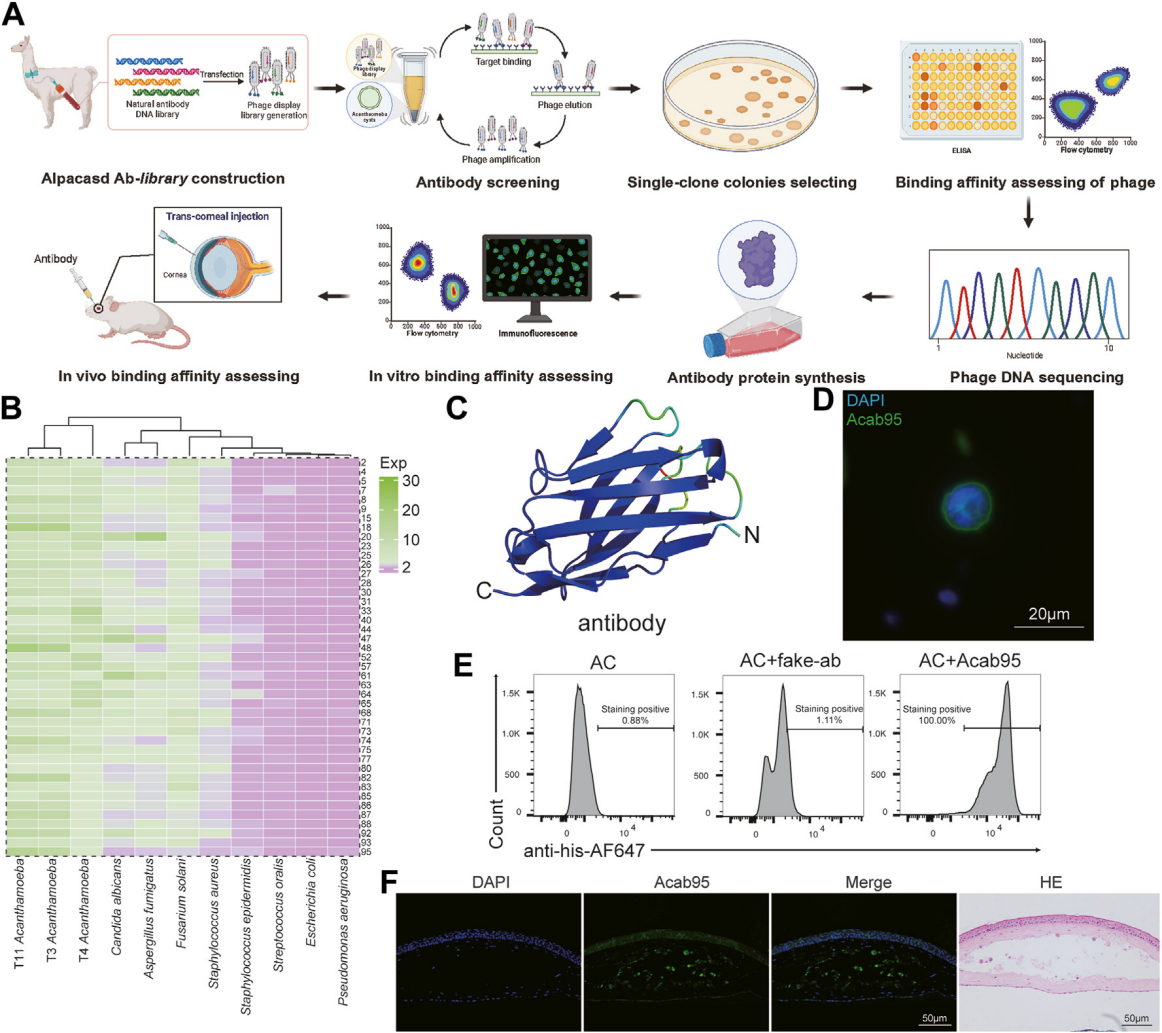
Screening amebic nano-antibodies and validation of their affinity *in vitro* and *in vivo* assays. (A) The experimental workflow for the screening and validation of nano-antibodies (Figure drawn using BioRender, NO. PA281UU7ZP). (B) Phage ELISA results demonstrating the specificity of the developed nano-antibodies against commonly encountered ocular pathogens. Green indicates high affinity and purple indicates low affinity. (C) Structural snapshots of antibody generated by AlphaFold2. (D) Immunofluorescence labelling demonstrating the binding of antibody protein to the cyst wall between *in vitro* experiments. (E) Flow cytometry analysis demonstrating the binding ability of the antibody to *Acanthamoeba*. (F) Immunofluorescence labelling illustrating the *in vivo* binding of antibody proteins.

To effectively deliver CXCL1 to the deep corneal stroma surrounding the *Acanthamoeba*, we would like to fuse CXCL1 to *Acanthamoeba*-specific antibodies to enhance targeted delivery. Initially we synthesized the MTAC4 antibody that was reported to recognize *Acanthamoeba* referenced in the literature^25^; however, we found it had no significant affinity for clinical *Acanthamoeba* strain^25^ (Supplementary Fig. S5). To identify high affinity antibodies recognizing the surface of *Acanthamoeba*, we hypothesized that it might be existed in the natural antibody repertoire of alpacas, the animals are commonly encountered the water sources with *Acanthamoeba*. Therefore, we utilized a naïve antibody library derived from B cells of alpacas to rapidly identify specific antibodies against *Acanthamoeba*. Three screening rounds were performed to isolate the most promising *Acanthamoeba*-specific antibodies by obtaining active protein-binding clones from the enriched library (Fig. 4A). Subsequently, ninety-six randomly selected colonies were tested for specific protein-binding with T4 *Acanthamoeba* using a phage ELISA. Out of these, 93 positive clones were selected for genomic sequencing, and 79 clones successfully sequenced (Supplementary Table S2). After removing duplicates (Supplementary Table S3), 43 unique clones remained. Their binding specificity against other *Acanthamoeba* genotypes, as well as bacteria and fungi commonly associated with eye infections, was tested using a phage ELISA. Fig. 4B demonstrates that most phages exhibited affinity with *Acanthamoeba* and fungi, but not bacteria. However, phage clone 95 demonstrated specific binding affinity only for *Acanthamoeba* and was therefore selected for further evaluation.

Using the open-source AlphaFold2 for protein tertiary structure prediction, we identified a structure with high confidence (Fig. 4C). Then, we produced recombinant VHH antibodies with N-terminally 6 × His-tagged in *E. coli* strain BL21(DE3). Coomassie-stained SDS-PAGE verified the presence of recombinant protein at the molecular weight of approximately 15 kDa (Supplementary Fig. S6). The binding specificity of Acab95 was evaluated through immunofluorescence staining and flow cytometry, which revealed that Acab95 recognized the surfaces of *Acanthamoeba* cyst walls (Fig. 4D) and exhibited excellent binding affinity (Fig. 4E). Additionally, immunofluorescence staining of the cornea demonstrated that Acab95 retained its binding capability *in vivo* (Fig. 4F). These results confirm the successful identification of an anti-*Acanthamoeba* antibody derived from a naïve antibody library of alpacas.

### Construction of Acab95-CXCL1 fusion protein and therapeutic efficacy validation

**Fig. 5:**
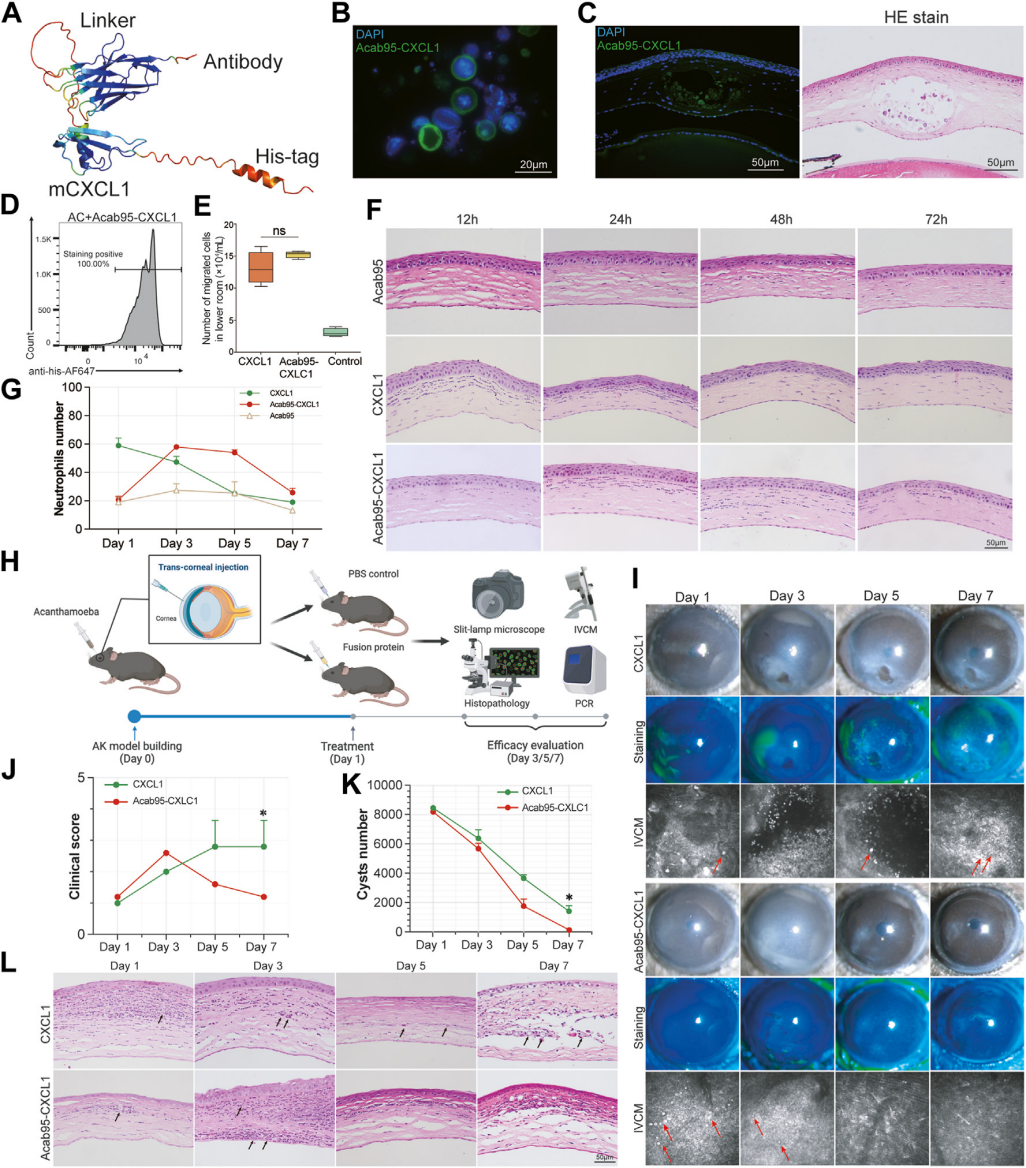
Evaluation of the binding ability, chemotactic ability and therapeutic efficacy of chemokine-nanobody fusion proteins. (A) Structural snapshots of chemokine-nanobody fusion proteins generated by AlphaFold2. (B and C) Immunofluorescence labelling demonstrating the binding of the chemokine-nanobody fusion proteins to the cyst wall *in vitro* and *in vivo* experiments. (D) Flow cytometry (FACS) analysis demonstrating the binding ability of the chemokine-nanobody fusion proteins to *Acanthamoeba*. (E) Evaluation of the chemotactic abilities of nanobody, chemokine and chemokine-nanobody fusion proteins using *in vitro* (n = 3 per group, n = 9 total; t-test). (F and G) Evaluation the chemotactic abilities *in vivo* and quantification of neutrophils number (n = 3 for each time point, n = 36 total). (H) The experimental workflow for evaluating therapeutic efficacy *in vivo* (Figure drawn using BioRender, NO. PJ281UUMZ4). (I) Slit-lamp microscope images with and without fluorescein staining, and IVCM images of mice at different days post-treatment. (Red arrow: *Acanthamoeba* cysts). (J) Clinical scoring of corneal infection. (n = 3 per group, n = 6 total; t-test) (K) Quantification of *Acanthamoeba* over time during treatment using real-time PCR. (n = 4 for each time point, n = 32 total; t-test) (L) HE stains of corneal sections from each group, assessed 1-, 3-, 5- and 7-day post-treatment (n = 3 for each time point, n = 24 total; Black arrow: *Acanthamoeba* cysts). (∗ for *P* < 0.05, ∗∗ for *P* < 0.01, ∗∗∗ for *P* < 0.001).

To facilitate the delivery of chemokines into the deep layer of the cornea, we designed a CXCL1 protein fusion with Acab95. Structures prediction using AlphaFold2 showed high confidence in the design of the Acab95-CXCL1 fusion protein (Fig. 5A). The highly purified Acab95-CXCL1 fusion protein, expressed in a prokaryotic system, migrated at approximately 23 kDa in SDS-polyacrylamide gels (Supplementary Fig. S6). The high affinities and binding site of the fusion proteins for *Acanthamoeba* were determined by *in vitro* and *in vivo* experiments using immunofluorescence staining (Fig. 5B and C). Flow cytometry showed the high fusion protein-binding affinity, which was consistent with the value of antibody (Fig. 5D).

Furtherly, transwell migration assay of neutrophils showed no differences in chemotactic responses to CXCL1 and Acab95-CXCL1 fusion protein (Fig. 5E). To validate the chemotactic ability *in vivo*, we injected Acab95, CXCL1, and the Acab95-CXCL1 fusion protein separately into the mouse cornea. At all-time points, cornea slides from the group treated solely with the Acab95 antibody exhibited no significant neutrophil infiltration. In contrast, the group injected with the CXCL1 chemokine alone demonstrated intense neutrophil infiltration at 12- and 24-h post-injection, followed by a notable decrease in the number of migrated neutrophils. In comparison to the individual components, the Acab95-CXCL1 fusion protein exhibited a slower yet more sustained chemotactic ability. At 12- and 24-h post-injection, neutrophil infiltration gradually increased, peaking at 48 h. Cornea slides continued to show evidence of neutrophil infiltration up to 72 h (Fig. 5F and G). These results indicate the Acab95-CXCL1 fusion protein contains both of the affinity of the antibody and the chemotactic properties of the chemokines.

The *in vivo* therapeutic efficacy of Acab95-CXCL1 fusion protein was evaluated by AK mouse model, as illustrated in the flowchart (Fig. 5H). Slit-lamp microscopy and fluorescein staining revealed the ongoing inflammatory reaction and corneal damage in CXCL1-treated group, while IVCM showed the persistent presence of *Acanthamoeba* cyst (Fig. 5I). In contrast, the group treated with the fusion protein showed significant inflammation by day 3, but by day 7, there was nearly complete normalization of the cornea and resolution of corneal oedema. In terms of IVCM detection, *Acanthamoeba* cysts were observed only on days 1 and 3, with none detected on days 5 and 7. Additionally, the fusion protein-treated group showed increased clinical score on day 3 but significantly decreased by day 7 (Fig. 5J).

To confirm these findings, absolute quantitative PCR was performed, revealing a decrease in cysts number in both groups, with the fusion protein-treated group achieving pathogen clearance by day 7 (Fig. 5K). Histopathological findings supported these results. In the CXCL1-treated group, cornea slides exhibited significant infiltration of neutrophils on the first day, which gradually decreased thereafter. However, *Acanthamoeba* cysts remained present throughout in the deep layer. In contrast, the peak of inflammation was on the third day in the fusion protein-treated group. Neutrophils only covered the area of cysts. Then the inflammation subsided rapidly, with no *Acanthamoeba* cysts detected in any corneal layer (Fig. 5L). These findings demonstrated that the Acab95-CXCL1 fusion protein exhibited excellent targeting and effectively cleared the pathogens from the deep layers.

## Discussion

Infectious diseases, such as hepatitis, tuberculosis, AIDS, meningitis, amoebiasis and so on, constitute major global health burdens, resulting in millions of deaths each year.^26^, ^27^, ^28^, ^29^, ^30^, ^31^, ^32^ Among these, parasitic infection is one of the most intractable problems of infectious diseases, and still understudied in terms of pathogen-host interactions and immune response mechanisms.^33^^,^^34^ This gap in research has limited the development of effective treatments. Through single-cell transcriptome analysis, we identified a unique immune cell landscape with decreased neutrophil counts and chemokine deficiency in AK patients. In response, we developed a chemokine-based immunotherapy strategy. Based on it, we employed anti-*Acanthamoeba* antibodies to guide chemokines, leading to favourable clinical outcomes. Now, there is still a lack of relevant studies addressing the immunotherapies of parasitic infections. Our cytokine-antibody approach provided a promising therapeutic strategy for treating parasitic diseases.

Although scRNA sequencing data on keratitis or Amoeba infections remains limited, several studies examining the immune response to other parasitic infections, such as malaria,^35^ *Heligmosomoides polygyrus*,^36^ *Clonorchis sinensis*,^37^ *Echinococcus granulosus*,^38^ *Toxoplasma gondii*.^39^ Similar to our research, the landscape of malaria or *T. gondii* infection also revealed an immunosuppressive environment with the presence of special immunosuppressive cells. And the studies also revealed distinct mechanisms of immune evasion and decreased chemokine expression. For example, previous research has shown that certain inhibitory immune cell populations, like SPP1^+^ macrophages, can promote immune evasion by suppressing T cell receptor expression in *E. granulosus* infection.^38^ Similarly, studies on malaria have identified interleukin (IL)-10-producing regulatory B cells as a major tolerogenic response, accompanied by decreased levels of key cytokines like TNF, IL-1β, and IL-6, and the chemokine MCP-1.^35^ Additionally, research on *T. gondii* infection has revealed the mechanism by which this pathogen can induce host cells to release paracrine factors that inhibit inflammatory gene transcription.^40^ *T. gondii* can also use the rhoptries effectors to degrade NF-κB, thereby decreasing the production of cytokines like IL-6, IL-12, and TNF.^41^ In our study, we observed a reduced proportion of neutrophils and significant heterogeneity within the neutrophil population on corneal tissues from AK patients. Importantly, the expression of chemokines involved in neutrophil chemotaxis, such as CXCL1, CXCL8, as well as the cytokine TNF, was also decreased in AK patients compared to non-AK group.

The observed immune evasion and chemokines deficiency suggest the potential for supplementing chemokines to promote neutrophil migration. Indeed, cytokine therapy has made significant strides toward clinical application.^42^^,^^43^ IL-2 was the first efficacious immunotherapy.^44^ However, its use has been limited by dose-related toxicities.^45^ Similarly, while IL-12 can stimulate immune activation and tumour control, the necessary doses can lead to severe adverse effects.^46^ Other interleukins,^40^, ^41^, ^42^, ^43^, ^44^, ^45^, ^46^, ^47^ such as IL-15, IL-18, IL-23, still required further investigation to confirm their therapeutic effects. The dose-limiting toxicities highlighted a need for improved targeting strategies.^48^

Targeted delivery methods that focus on achieving high local concentrations of cytokines at the site of lesions, while minimizing systemic exposure, have revitalized interest in cytokine therapy.^49^, ^50^, ^51^, ^52^, ^53^ Recently, Santollani et al.^54^ designed two types of cell surface-targeted ‘immunocytokines’, αCD45-IL-12 and αCD45-IL-15. A single intra-tumoral injection of these ‘immunocytokines’ successfully eradicated both treated tumours and untreated distal lesions in multiple syngeneic mouse tumour models without causing toxicity. However, not all antibody-cytokine fusion proteins function effectively. The study of Tzeng et al.^55^ found that the cytokine component could dominate over antibody-mediated targeting, leading to localization in cytokine receptor-expressing cells rather than the intended target cells. While there have been no prior studies utilizing antibody-cytokine fusions for parasitic infections, the similar situation of immune evasion suggests that ‘immunocytokines’ could be a promising avenue for exploration. Moreover, the substantial gaps between pathogens and their hosts may reduce off-target binding risks, and the lower densities of immune cell infiltration in parasite decreases the likelihood of cytokine receptor-expressing cell targeting.^56^, ^57^, ^58^ Particularly, the cornea was an extensive immune-privileged site and tissue.^59^ This unique superiority allows to apply this intervention. Our results support this approach: the fusion protein was able to effectively target and clear *Acanthamoeba* in the deep layers of the cornea. This approach is likely applicable not only for AK, but also for other parasitic infections that have immunosuppressive characteristics. Compared with direct cytokine therapy, 'immunocytokines' could decrease dose-limiting toxicities and extending their activity *in vivo*.

However, our study still has several limitations that will need to be improved. First, this study was conducted at a single tertiary hospital, resulting in enrolment of patients with severe clinical presentations. This may limit the generalizations of the findings. Second, the current mode of administration is via intrastromal injection, which may not be the most practical for clinical application. In future research, we aim to explore alternative drug delivery methods, such as using eye drops, to enhance the convenience and feasibility. Third, the data presented here are based solely on experiments conducted in mouse models. Before advancing to clinical trials, it is crucial to validate our findings in primate models to ensure consistency with human immune responses. At that time, the chemokine-antibody fusion proteins will need to be further optimized to ensure optimal compatibility with the human immune system. Additionally, the sample size is too small. This may explain why most of the cell subpopulations didn’t show statistical differences. We expect obtained more samples to further validate these discoveries in the next steps.

In summary, *Acanthamoeba* keratitis, as a parasitic disease, is characterized by heterogeneity in neutrophil count and function. The observed immune evasion mechanisms and chemokine deficiencies in this condition suggest that targeted delivery of immunocytokines could be an attractive precision medicine approach. Unlike traditional cytokine therapy, these chemokine-antibody fusion proteins can potentially overcome dose-limiting toxicities and extend their activity *in vivo*, while specifically targeting parasites in the deep corneal layers. The promising results from this study warrant further optimization and validation in primate models. This precision immunotherapy approach takes into account the unique immunological characteristics of parasitic diseases and holds great promise for enhancing treatment of AK, as well as potentially addressing other infectious diseases characterized by immune evasion.

## Contributors

Z.W. conceived, designed, and performed the analysis, analysed the results. J.Y. analysed the flow cytometry results and constructed the plasmid. Q.C. and J.P. reviewed the analysis. Q.S. and B.P. reviewed the methodology. M.W. and Y.W. managed animal experiments. Z.Z. provided the histology images. X.L. provided evaluation of laboratory data of the patients. X.L. provided the antibody screening platform. The manuscript was written by Z.W., X.L. and Q.L. All authors read and approved the final submitted manuscript. Xin Lin and Qingfeng Liang were co-corresponding authors for this work. The corresponding authors, X.L. and Q.L., verified all underlying data and took final responsibility for the decision to submit for publication.

## Data sharing statement

scRNA-seq data are publicly available in CNGB: CNP0006471. Any additional information required to reanalyse the data reported in this paper is available from the lead contact upon request.

## Declaration of interests

The authors declare that they have no competing interests.
