# Supplementary material for: Bifunctional chemokine-nanobody fusion protein enhances neutrophil recruitment to impede Acanthamoeba immune evasion
Source: eBioMedicine. 2025 Apr 12;115:105685. doi: 10.1016/j.ebiom.2025.105685 (PMC12013128; doi:10.1016/j.ebiom.2025.105685)
Supplement: Supplementary Figs. S1–S6 [file mmc1.docx]

**

**

**Supplemental figure 1.** The gene heatmap and GO enrichment analysis results for major cell cluster.

**Supplemental figure 2.** Correlation between samples was calculated using Pearson’s correlation coefficient.


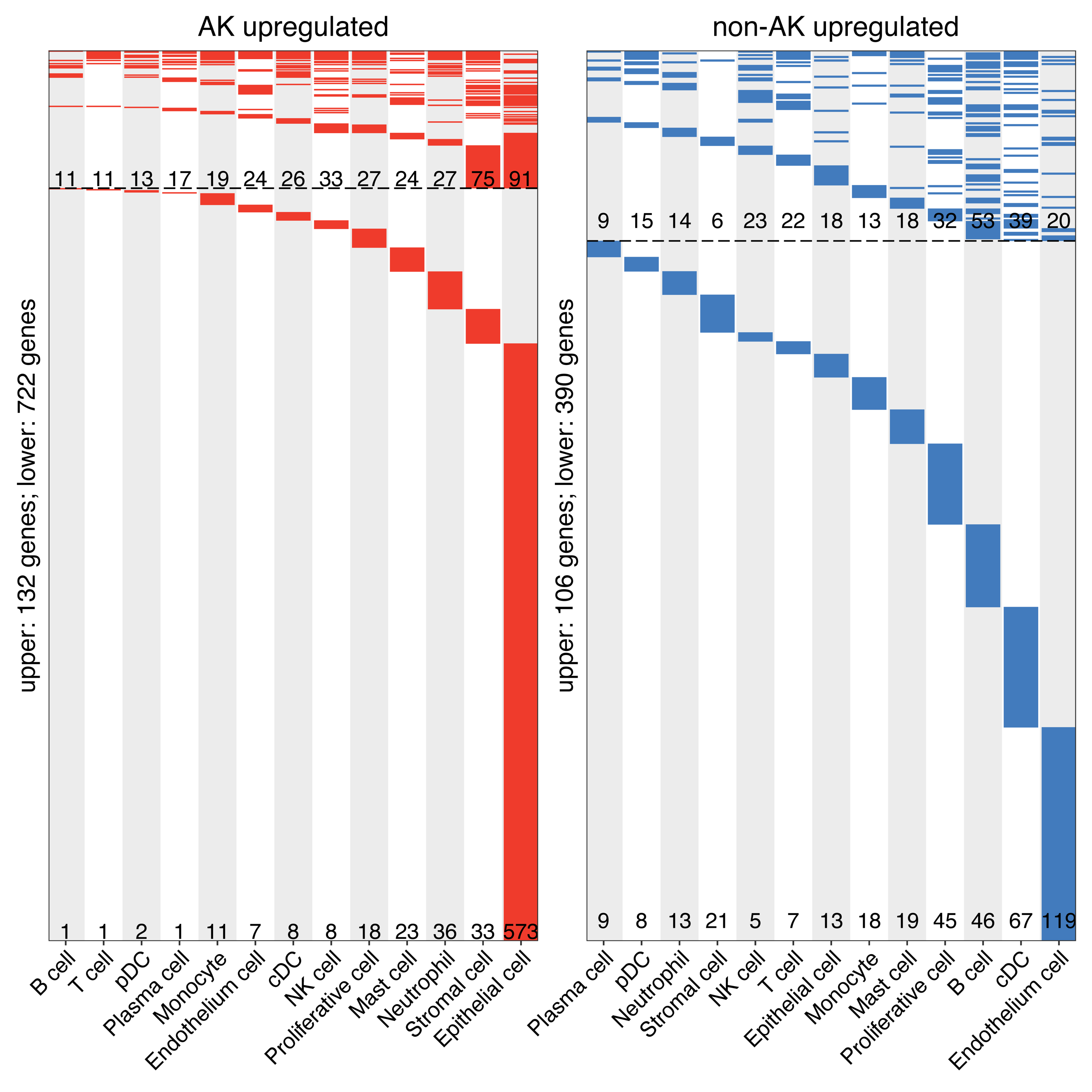


**Supplemental figure 3.** The differential genes of cell subsets between AK group and non-AK group.

**Supplemental figure 4.** The GO and KEGG pathway enrichment analysis of neutrophil in AK group and non-AK group.


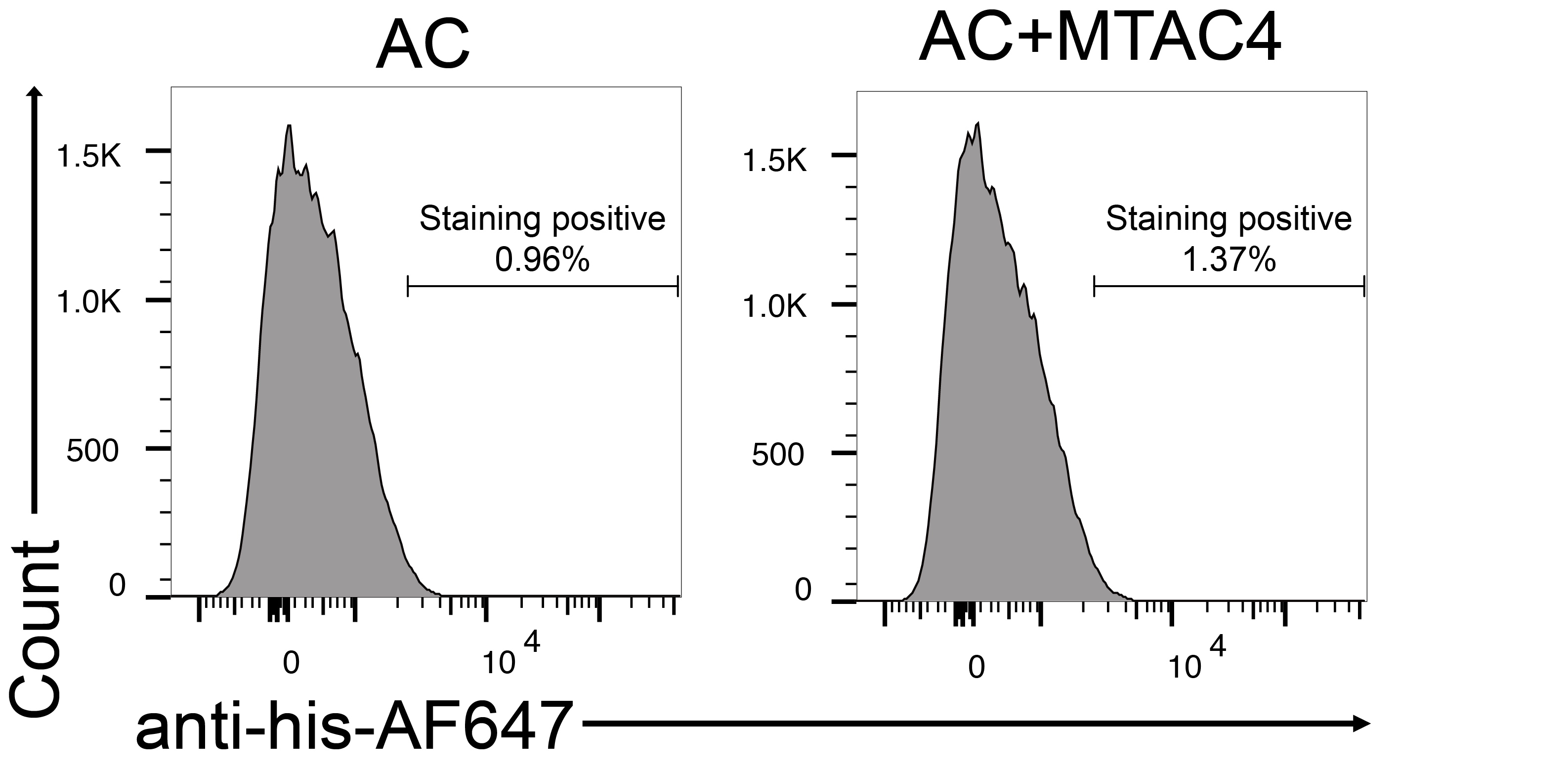


**Supplemental figure 5.** Evaluation of the binding ability of antibody MTAC4, which were obtained from literature.


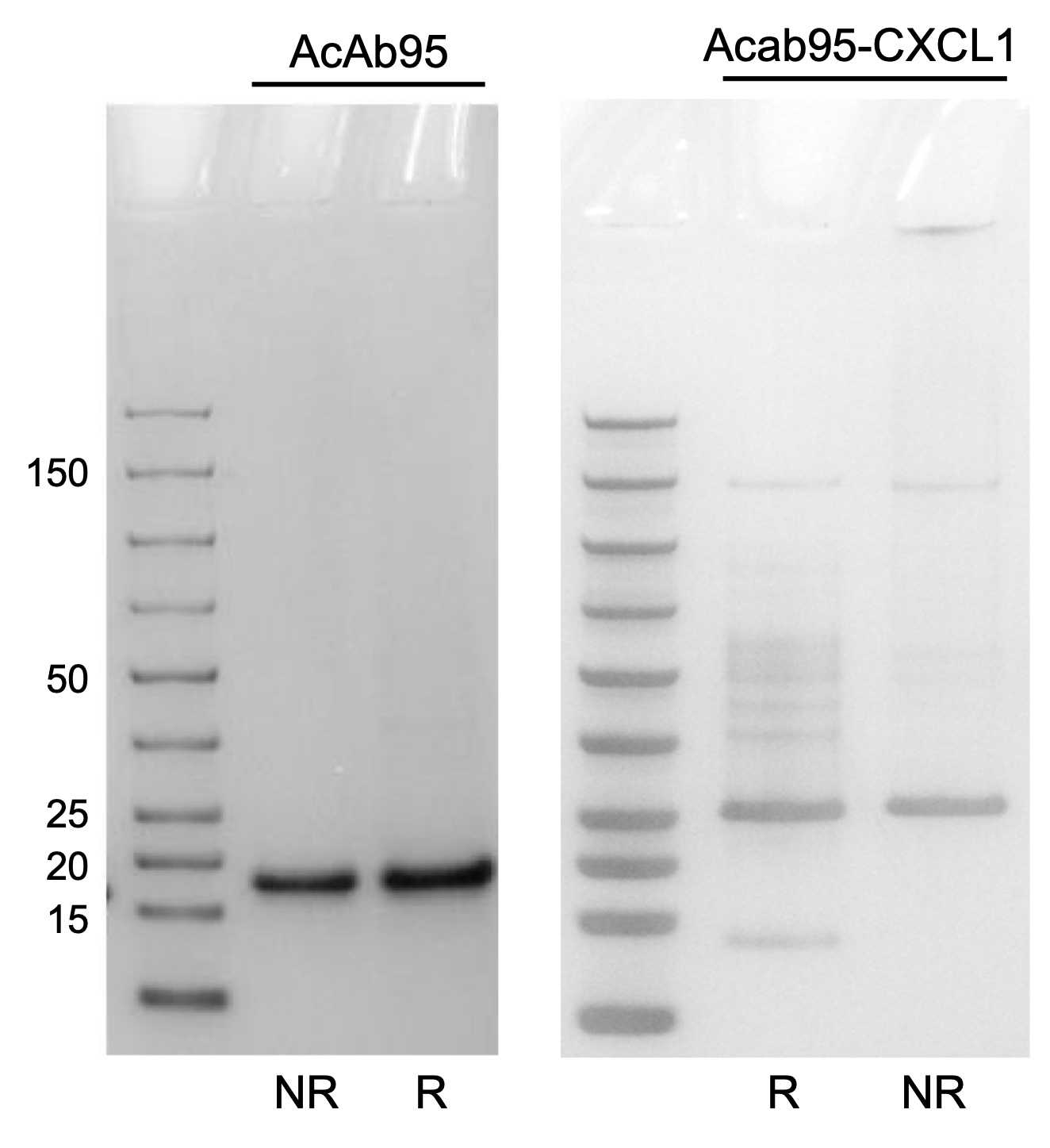


**Supplemental figure 6.** Expression of Acab95 and Acab95-CXCL1 fusion protein using the *E. coli* system. (NR: non-reducing conditions, R: reducing conditions)
